# The relationship between athletic identity and kinesiophobia in young athletes with prior sport-related injury

**DOI:** 10.3389/fspor.2026.1836002

**Published:** 2026-06-24

**Authors:** Antonia L. Dinulescu, Ava N. Davis, Emily B. Gale, Emily J. Stapleton, Joseph Janosky, Henry B. Ellis, Sophia Ulman

**Affiliations:** 1 Scottish Rite for Children, Frisco, TX, United States; 2 The University of Texas Health Houston, McGovern Medical School, Houston, TX, United States; 3 University of Texas Southwestern Medical Center, Dallas, TX, United States; 4 Lasell University, Newton, MA, United States

**Keywords:** adolescent, identity formation, rehabilitation, reinjury, return to sport, sports psychology

## Abstract

**Objectives:**

Athletic identity, the degree to which an individual identifies with the athlete role, and kinesiophobia, the fear of reinjury, are both psychological factors that play a role in a young athlete's performance and injury rehabilitation. The purpose of this study was to evaluate the relationship between athletic identity and kinesiophobia in young athletes with recent sports-related injury.

**Methods:**

A convenience sample of 220 participants completed a series of surveys including the Athletic Identity Measurement Scale (AIMS) and the Tampa Scale for Kinesiophobia (TSK-11). Data analysis included Mann–Whitney U tests and Spearman correlations.

**Results:**

A total of 220 young athletes with a recent sports-related injury (52.7% female; 14.6 ± 2.0 years; 8.3 ± 3.0 years active in sport) were included for analysis. The majority of participants played soccer (42.8%), basketball (25.2%), and baseball/softball (11.3%) and had a high sport specialization level (64.9%). AIMS total score was 54.70 ± 8.77 (range: 8–70), indicating a relatively strong athletic identity. TSK-11 total score was 19.27 ± 5.82 (range: 11–44), indicating slight kinesiophobia. No significant sex differences were found; however, significant relationships were found between TSK-11 score and AIMS subscales as well as between AIMS subscales. TSK-11 total score demonstrated weak but statistically significant positive correlations with age, exclusivity, and negative affectivity. In regard to AIMS subscales, there was a weakly positive correlation between exclusivity and social identity, but a moderately positive correlation between exclusivity and negative affectivity.

**Conclusions:**

These results indicate that athletes with a stronger athletic identity may suffer psychological consequences that could have clinical implications on injury rehabilitation. Future work should seek to validate the current study's findings and identify other psychosocial factors involved in this relationship.

## Introduction

Psychological factors play a major role in a young athlete's career development and injury rehabilitation. Among them is the concept of athletic identity, defined by Brewer et al. as the degree to which an individual identifies with the athlete role (4). Current research has shown that a strong athletic identity can be both favorable and unfavorable for an athlete (8). During athletic development, a stronger athletic identity is associated with increased athlete satisfaction, physical activity, sports team participation, and years active in a sport (1, 21). With exposure to an active and collaborative environment, athletic identity thus encourages the maintenance of a large social network and a stronger social identity (5, 24, 26).

Although a strong athletic identity can have a positive impact on a developing athlete, the occurrence of an injury may jeopardize an athlete's identity. Over the course of post-operative anterior cruciate ligament (ACL) rehabilitation, athletic identity was found to significantly decrease as athletes reduced their identification with their athletic status (3, 20). Athletes with a strong athletic identity exclusive to a single sport also demonstrate increased depressive symptom severity, emotional disturbance, and difficulty adjusting to sport cessation post-injury postulated in part due to the loss of their strong social network (18, 25, 31). With these consequences in mind, it is important to develop a more informed understanding of the psychosocial impact of athletic identity (8).

The effect of injury on athletic identity could potentially play a role in the development of kinesiophobia, systematically defined as the fear of movement but commonly referred to in sports medicine literature as the fear of reinjury (6, 15). Elevated levels of kinesiophobia are strongly associated with prolonged injury recovery, decreased performance and muscle strength, and greater re-injury rates (6, 15, 30). Kinesiophobia also affects an injured athlete's psyche through increased fear avoidance, pain catastrophizing, and emotional distress (10). For these reasons, kinesiophobia has been established as the leading reason for failure to return to sport in athletes (27, 30). Although kinesiophobia is well-studied in young athletes, its relationship to athletic identity has not been evaluated to the best of our knowledge. For this reason, our study aims to assess this relationship in healthy adolescent athletes with a recent sport-related injury to allow for more informed clinical practice. We hypothesized that this assessment would reveal a positive relationship between athletic identity and level of kinesiophobia.

## Methods

### Participants

A convenience sample of 220 athletes between 8 and 18 years old with any sport-related injury within the past 12 months were asked to complete electronic surveys as part of a community-based research study from October 2022 to July 2024. Participants without a recent injury and not actively engaged in sport were excluded from the study. This study was approved by the local Institutional Review Board (#STU-082010-134), and thus, participants provided informed assent and/or consent prior to completing study procedures.

### Procedures

Surveys were administered in person on a tablet device. Demographics collected included age, sex, and primary sport. Level of sport participation was inferred from questions inquiring about time dedicated to organized sport, competition level(s), and whether the athlete identified as a multi- or single-sport athlete. Additionally, to quantify specialization status, participants were asked whether they (1) felt they had a ‘main sport,’ (2) quit other sports to focus on that main sport, and (3) trained in that main sport for more than eight months in a single year. For each “no” response, a value of 0 was assigned and for each “yes” response a value of 1 was assigned. The sum of the positive responses was interpreted as low (total of 0 or 1), moderate (total of 2), and high specialization (total of 3) (16). Additionally, responses to the Athletic Identity Measurement Scale (AIMS) and the Tampa Scale for Kinesiophobia (TSK-11) were collected (4, 32). Notably, the TSK-11 was only collected from participants who reported at least one sports-related injury in the past 12 months. Details on the recent injury were not consistently captured as the more detailed injury history questions were optional.

### Instruments

Developed by Brewer et al. in adult populations, the AIMS is a 10 statement questionnaire that uses a seven-point Likert scale (1 = strongly disagree, 7 = strongly agree) to measure the extent to which a participant identifies with their athlete role (4). The sum of all 10 responses yields the total AIMS score, with higher scores delineating a stronger athletic identity. Athletic identity thresholds have not been validated yet, but prior works involving similar young athlete populations have used 50 as the threshold for a high athletic identity (21, 25). For the purposes of this study and for continuity, the threshold of 50 was maintained despite the lack of validation. The AIMS total score can be broken down into three subscale scores for social identity (questions 1–3), exclusivity (questions 4–6 and 9), and negative affectivity (questions 8 and 10). The social identity factor measures the extent to which an athlete's self-concept is derived from membership to a social group (4). The exclusivity factor measures the predominance of an athlete's athletic identity over others (4). The negative affectivity factor measures the participant's emotional response to time away from sport or undesirable sport outcomes (21). The AIMS has been established in literature with good reliability and internal consistency (2, 4), and has been utilized for analysis of athletic identity in young athletes (20, 21).

The TSK-11 is an 11-item questionnaire designed to assess kinesiophobia, the fear of physical movement or activity that can lead to re-injury (11, 32). Each question is scored on a four-point Likert scale, with responses ranging from 1 (strongly disagree) to 4 (strongly agree) (32). The TSK-11 total scores can range from 11 to 44, with higher scores indicating greater kinesiophobia. Scores are divided into the following thresholds: no fear (0–17 points), slight fear (18–24 points), moderate fear (25–31 points), severe fear (32–38 points), and maximum fear (39–44 points). The TSK-11 was chosen as a measure for kinesiophobia due to its excellent internal consistency (Cronbach's *α* = 0.79) and good reliability (ICC = 0.81) in analysis involving young adults (11, 32).

### Data analysis

Means and standard deviations were calculated for all continuous variables, including age, time in sport (years), AIMS subscales and total score, and TSK-11 total score. Given significant Shapiro–Wilk tests for normality, Mann–Whitney U tests were conducted to identify significant sex differences in AIMS subscales and total score and TSK-11 total score. Effect size *r* was calculated to quantify the magnitude of between-group differences independent of sample size. Additionally, Spearman correlations were conducted to identify relationships between measures. Correlation coefficients were classified as weak (*r* < 0.3), moderate (*r* = 0.3–0.7), or strong (*r* ≥ 0.7) (12). 95% confidence intervals were also computed. All analyses were performed using SPSS Statistics (IBM SPSS Statistics for Windows, version 224.0, Armonk, NY, USA).

## Results

A convenience sample of 220 young athletes with any recent sport-related injury in the past 12 months (52.7% female; 14.6 ± 2.0 years; 8.3 ± 3.0 years active in sport) was included for analysis. The primary sport and specialization level across participants are provided in Table 1. Lower extremity injuries were notably the most common among participants that chose to disclose injury type. On average, AIMS total score was 54.70 ± 8.77 (range: 8–70), indicating a relatively strong athletic identity. On average, TSK-11 total score was 19.27 ± 5.82 (range: 11–44), indicating slight kinesiophobia. No significant sex differences were found in TSK-11 total score (*p* *=* 0.148) or any AIMS subscales or total score (*p* > 0.05; Table 2).

**Table 1:** Sport characteristics of participants.

Primary sport	*N*	%
Basketball	56	25.2
Soccer	95	42.8
Gymnastics	20	9.0
Baseball/Softball	25	11.3
Volleyball	15	6.8
Other	11	5.0

Specialization level	*N*	%
Low	16	7.2
Medium	62	27.9
High	144	64.9

‘Other’ sports included track, football, golf, lacrosse, and dance.

**Table 2:** TSK-11 total score and AIMS scores by sex.

Variable	Male	Female	*p*-value	Effect size (*r*)
Social Identity	19.69 ± 2.76	19.63 ± 2.04	0.163	−0.09
Exclusivity	18.54 ± 4.96	17.73 ± 5.04	0.151	−0.10
Negative Affectivity	10.31 ± 2.91	10.22 ± 3.23	0.863	−0.01
AIMS Total Score	54.00 ± 9.48	53.42 ± 8.20	0.298	−0.07
TSK-11 Total Score	18.71 ± 6.16	19.77 ± 5.49	0.148	−0.10

Values presented as mean ± standard deviation.

Significant relationships were found between TSK-11 score and AIMS subscales. TSK-11 total score demonstrated weak but statistically significant positive correlations with age, exclusivity, and negative affectivity (Table 3). Specifically, higher TSK-11 scores were associated with older age, *r* = 0.14, *p* = 0.041, 95% CI [0.00, 0.27]; greater exclusivity, *r* = 0.15, *p* = 0.027, 95% CI [0.01, 0.28]; and greater negative affectivity, *r* = 0.19, *p* = 0.006, 95% CI [0.05, 0.03]. These findings suggest that greater kinesiophobia was modestly related to being older and to stronger athletic identity-related exclusivity and negative emotional responses. TSK-11 total score was not significantly associated with total years active in sport, social identity, or total AIMS score.

**Table 3:** Spearman correlation coefficients to identify relationships between measures.

Measure	Years active	Social identity	Exclusivity	Negative affectivity	AIMS total	TSK-11 total
Age	**0.52** **[0.41, 0.61]** ***p*** **<** **0.001**	0.11 [−0.02, 0.24] *p* = 0.097	0.05 [−0.08, 0.19] *p* = 0.423	0.02 [−0.12, 0.15] *p* = 0.793	0.07 [−0.07, 0.20] *p* = 0.333	**0.14** **[0.00, 0.27]** ***p*** **=** **0.041**
Years Active	–	0.07 [−0.07, 0.20] *p* = 0.295	0.02 [−0.12, 0.16] *p* = 0.771	0.05 [−0.09, 0.19] *p* = 0.451	0.06 [−0.08, 0.19] *p* = 0.399	0.01 [−0.13, 0.14] *p* = 0.945
Social Identity	–	–	**0.21** **[0.08, 0.24]** ***p*** **=** **0.001**	−0.04 [−0.17, 0.10] *p* = 0.590	**0.35** **[0.22, 0.46]** ***p*** **<** **0.001**	−0.07 [−0.21, 0.06] *p* = 0.273
Exclusivity	–	–	–	**0.51** **[0.40, 0.60]** ***p*** **<** **0.001**	**0.89** **[0.86, 0.91]** ***p*** **<** **0.001**	**0.15** **[0.01, 0.28]** ***p*** **=** **0.027**
Negative Affectivity	–	–	–	–	**0.70** **[0.62, 0.76]** ***p*** **<** **0.001**	**0.19** **[0.05, 0.31]** ***p*** **=** **0.006**
AIMS Total	–	–	–	–	–	0.12 [−0.02, 0.25] *p* = 0.075

Statistically significant correlations noted in bold.

Significant relationships were also found between AIMS total score and AIMS subscales, which was to be expected, as well as between AIMS subscales (Table 3). There was a weakly positive correlation between exclusivity and social identity, *r* = 0.21, *p* = 0.001, 95% CI [0.08, 0.24]; and a moderately positive correlation between exclusivity and negative affectivity, *r* = 0.51, *p* < 0.001, 95% CI [0.40, 0.60]. These findings suggest that membership to a social group and negative emotional response to time away from sport or an undesirable sport outcome were related to a greater predominance of athletic identity over other identities. In addition, there was also a moderately positive correlation between years active and age, *r* = 0.52, *p* < 0.001, 95% CI [0.41, 0.61], confirming that older participants spent more years active in their sport.

## Discussion

To better understand the psychosocial impact of athletic identity on a young athlete's career development and injury rehabilitation, the current study aimed to evaluate the relationship between athletic identity (AIMS) and kinesiophobia (TSK-11) in young athletes with a recent sport-related injury. We hypothesized that our assessment would demonstrate a positive relationship between athletic identity and level of kinesiophobia. Although there was no significant relationship between AIMS total score and TSK-11 total score as well as no sex differences, this study's primary findings were significant positive associations between TSK-11 score and AIMS subscales. TSK-11 total score demonstrated weak but statistically significant positive correlations with age, exclusivity, and negative affectivity. These findings suggest that greater kinesiophobia was modestly related to being older and to stronger athletic identity-related exclusivity and negative emotional responses. This study's secondary findings were significant positive associations between AIMS subscales. Social identity demonstrated a weak positive correlation with exclusivity while negative affectivity had a moderate positive correlation with exclusivity. These findings suggest that membership to a social group and negative emotional response to time away from sport or an undesirable sport outcome were related to a greater predominance of athletic identity over other identities.

This study's primary finding suggests that stronger athletic identity-related exclusivity and negative emotional responses were significantly associated with elevated levels of kinesiophobia, which could be explained by the unidimensional characteristic of an exclusive, strong athletic identity. Thoits demonstrated that having a unidimensional self-identity is associated with more psychological stress and is characterized by extreme emotional swings and frequent depression (29). Additionally, Linville found that self-complexity through multiple identities results in less psychological stress (17). Since a strong athletic identity is associated with increased exclusivity and sport specialization level (5), the positive association between athletic identity and kinesiophobia supports the claims that exclusive self-identification has negative psychological impacts, including elevated levels of depression and emotional trauma (18, 25). Increased kinesiophobia in athletes with a strong athletic identity could therefore be a by-product of the increased psychological stress, or negative affectivity, of an athlete dissociating from their sole source of identification during and after injury rehabilitation.

The study's finding of a positive association between age and kinesiophobia further justifies the argument that kinesiophobia could be a by-product of having a unidimensional, exclusive athletic identity. Previous literature has shown that young athletes tend to combine their athletic identity with their personality as they mature (8, 31), thus strengthening their athletic identity with increasing age (1, 8, 14). Recent trends have also demonstrated a shift towards earlier training and single sport specialization (7). Since the athletes surveyed were at the transition between early adolescence (10–15 years) and middle adolescence (15–18 years) (19), this literature supports the idea that their more exclusive identities, formed due to maturation and sports specialization, could be the reason for their elevated levels of kinesiophobia. However, literature explicitly analyzing the relationship between age and kinesiophobia is limited.

Another way to interpret the weakly positive association between athletic identity-related exclusivity/negative emotional responses and kinesiophobia is through a social lens. Studies have shown that sports participation in young athletes results in increased social integration, social well-being, social functioning, and overall better relationships with coaches and friends (9, 13, 28). As a result, athletes with a strong athletic identity tend to have a strong social identity (5, 24, 26). This tendency is supported by the finding of a weakly positive correlation between athletic social identity and athletic identity-related exclusivity in the current study. For these reasons, an alternative explanation for the positive association between athletic identity and kinesiophobia could be that kinesiophobia potentially stems from a fear of losing a strong social network once again.

Although the current study is the first to directly evaluate athletic identity and kinesiophobia in young athletes, some studies have analyzed associated psychosocial factors or evaluated this relationship indirectly with contradicting results. Ferman et al. studied fear avoidance perceptions and athletic identity in late adolescent physical therapy outpatients and found that athletes with a stronger athletic identity had weaker fear avoidance perceptions (9). Ohji et al. found that young adult athletes that returned to the same level of sport had a stronger athletic identity and sport commitment as well as lower levels of kinesiophobia (23). These findings deviate from the current study's results and could be explained by the later developmental stages of these athletes.

Other studies involving related psychosocial factors substantiate our findings. Specifically, Manuel et al. found that adolescent athletes with higher athletic identities had higher depression scores (18). Padaki et al. that young athletes with high athletic identities exhibited greater emotional trauma than those that had lesser athletic identities (25). Similarly, discusses athletic identity as an implicated re-injury risk factor, finding that those with high athletic identities were associated with subsequent injuries (22). Given the conflicting discourse, there is a clear need for supportive literature regarding the relationship between athletic identity and kinesiophobia.

### Limitations

Limitations of the current study include selection bias, a skewed athlete profile, lack of control for injury type, and causal inference. First, the use of a convenience sample may introduce selection bias and limits the generalizability of findings to a broader athletic population. Similarly, a majority of the young athletes surveyed in this study were highly specialized and participated in team sports; however, this study did not directly distinguish level of engagement and future intentions for the sport. This limits the generalizability of these results to other athletes including those participating in elite individual sports, recreational level activities, and international sport systems. The wide age range in our population without subgroup or stratified analysis also may mask developmental differences in athletic identity and fear responses as well as limits the generalizability given the differing athletic profiles and limited analysis. Similarly, this study didn't analyze or discuss cultural or sport-system differences, which could play a role in the study's findings. Furthermore, athletes were not required to disclose injury type or severity. Failure to control for injury characteristics such as type, severity, and chronicity represents a major confounder, as these variables are known to significantly influence kinesiophobia. Although this increased the heterogeneity in our young athlete population, it could aid in the generalizability of the study results. Finally, the cross-sectional, observational study design limits causal inference; therefore, results that suggest athletic identity infers increased kinesiophobia should be interpreted with caution. Future research should consider employing dimension reduction strategies to address these issues with a larger and more diverse cohort of young athlete. In addition, researchers should consider examining specific athlete profiles and developmental groups through both quantitative and qualitative approaches to further clarify the prevalence of kinesiophobia and its relation to athletic identity.

## Conclusion

The current study is the first to directly evaluate the relationship between athletic identity and kinesiophobia and establish its significant predictors. This study found that greater kinesiophobia was modestly related to being older and to stronger athletic identity-related exclusivity and negative emotional responses. Within athletic identity, this study found that membership to a social group and negative emotional response to time away from sport or an undesirable sport outcome were related to a greater predominance of athletic identity over other identities. Given the contradictory discourse in related literature, the negative consequences of kinesiophobia on a developing athlete, and study limitations, there is a justified need for a deeper understanding of the relationship between athletic identity and kinesiophobia to better treat young athletes undergoing injury rehabilitation in a clinical setting. Future studies should seek to validate the current study's findings while further exploring other psychosocial factors involved. Specifically, studies should consider longitudinal designs incorporating injury-specific variables and psychological mediators to better understand the temporal relationship between athletic identity and kinesiophobia.

## Data Availability

The raw data supporting the conclusions of this article will be made available by the authors, without undue reservation.
